# Axial stretch regulates rat tail collecting lymphatic vessel contractions

**DOI:** 10.1038/s41598-020-62799-x

**Published:** 2020-04-03

**Authors:** Mohammad S. Razavi, Julie Leonard-Duke, Becky Hardie, J. Brandon Dixon, Rudolph L. Gleason

**Affiliations:** 1 The George W. Woodruff School of Mechanical Engineering, Georgia Institute of Technology, 801 Ferst Dr., Atlanta, GA 30332 USA; 2 The Wallace H. Coulter Department of Biomedical Engineering, Georgia Institute of Technology, 313 Ferst Dr., Atlanta, GA 30332 USA; 3 The Parker H. Petit Institute for Bioengineering and Bioscience, Georgia Institute of Technology, 315 Ferst Dr., Atlanta, GA 30332 USA

**Keywords:** Biophysics, Medical research

## Abstract

Lymphatic contractions play a fundamental role in maintaining tissue and organ homeostasis. The lymphatic system relies on orchestrated contraction of collecting lymphatic vessels, via lymphatic muscle cells and one-way valves, to transport lymph from the interstitial space back to the great veins, against an adverse pressure gradient. Circumferential stretch is known to regulate contractile function in collecting lymphatic vessels; however, less is known about the role of axial stretch in regulating contraction. It is likely that collecting lymphatic vessels are under axial strain *in vivo* and that the opening and closing of lymphatic valves leads to significant changes in axial strain throughout the pumping cycle. The purpose of this paper is to quantify the responsiveness of lympatic pumping to altered axial stretch. *In situ* measurements suggest that rat tail collecting lymphatic vessels are under an axial stretch of ~1.24 under normal physiological loads. *Ex vivo* experiments on isolated rat tail collecting lymphatics showed that the contractile metrics such as contractile amplitude, frequency, ejection fraction, and fractional pump flow are sensitive to axial stretch. Multiphoton microscopy showed that the predominant orientation of collagen fibers is in the axial direction, while lymphatic muscle cell nuclei and actin fibers are oriented in both circumferential and longitudinal directions, suggesting an axial component to contraction. Taken together, these results demonstrate the significance of axial stretch in lymphatic contractile function, suggest that axial stretch may play an important role in regulating lymph transport, and demonstrate that changes in axial strains could be an important factor in disease progression.

## Introduction

Lymphatic vessels serve as the major route to transport lymph from the interstitial space to the great veins^1–3^. Collecting lymphatic vessels pump lymph against an adverse pressure gradient and have specialized lymphatic muscle cells (LMCs) that enable them to contract spontaneously and control their diastolic vascular caliber via tonic constrictions^3–5^. These rhythmic and synchronized contractions are orchestrated, with the help of one-way valves, to propel lymph efficiently along a chain of lymphangions^6,7^. Disruptions in this orchestrated function lead to impaired lymph transport and lymphedema.

Lymphatic vessels adapt their contractile function to various loading conditions (pressure, flow, and external forces from skeletal muscles) and this adaptation is important in their function both as a pump and as a conduit to efficiently transport lymph^8–12^. The effect of pressure-induced circumferential stretch on lymphatic contractile function has been well-documented^11,13–21^. At low circumferential stretches, increased circumferential stretch (due to increased transmural pressure) increases the lymphatic contraction frequency and results in an increase in the amplitude of contractions. At higher circumferential stretches, increased circumferential stretch decreases contraction amplitude^19,20,22^. Circumferential stretch is thought to regulate lymphatic contractility by increasing pace-making activity and calcium sensitivity^13^. Davis *et al*. has shown that LMCs generate a rate-sensitive response to stretch^23^ and Telinius *et al*. has shown human mesenteric lymphatics generate the maximum tension at a specific passive stretch corresponding to 22 mmHg^16^. A relationship between circumferential length and tension has been reported in lymphatic vessels from different tissues beds from humans, cows, and rats; however, the specific length-tension relationships vary depending on the tissue beds and animals under study^4,23–25^. Lymphatic muscles are very sensitive to changes in the level of intracellular Ca^2+^, and circumferential stretch has been implicated as a modulator of calcium sensitivity^14,16,26,27^. There is also evidence that electrical activity of lymphatic vessels change with changes in circumferential stretch; higher stretches induce Ca^2+^ sensitization that increases the responsiveness of key contractile molecules to Ca^2+^^28–30^.

Similar to circumferential stretch, axial stretch may also be a modulator of lymphatic function. In some regions, such as the lung and diaphragm, axial stretch changes due to applied external forces; e.g., pleural and diaphragm lymphatics can be exposed to cyclic stretches which enhance lymph formation and transport^31,32^. The effect of axial load on the phasic contractions has been studied via axial load-controlled experiments on the bovine mesenteric lymphatics^20^. McCale *et al*. showed that, in contrast to pressure-induced stretch that changed both the frequency and the strength of contractions, axial load only increased the strength of contractions while the frequency of contraction remained unchanged; further, it was observed that axial load linearly increased the axial stretch, under fixed pressures^20^. An increase in the axial load with both pressure and axial stretch has been reported for the rat thoracic duct^33^; however, there is no experimental evidence to show lymphatic vessels experience axial stretch *in situ*. Towards this end, the goal of this study is to quantify the *in situ* axial stretch of rat tail lymphatics and axial stretch-mediated modulation of lymphatic contractility in an *ex vivo* platform. Further, the distribution of collagen and LMCs’s of rat tail lymphatics in the circumferential-axial plane were quantified to understand the axial contributions of these structural constituents.

## Methods

### Animal model and vessel isolation procedure

**Figure 1:**
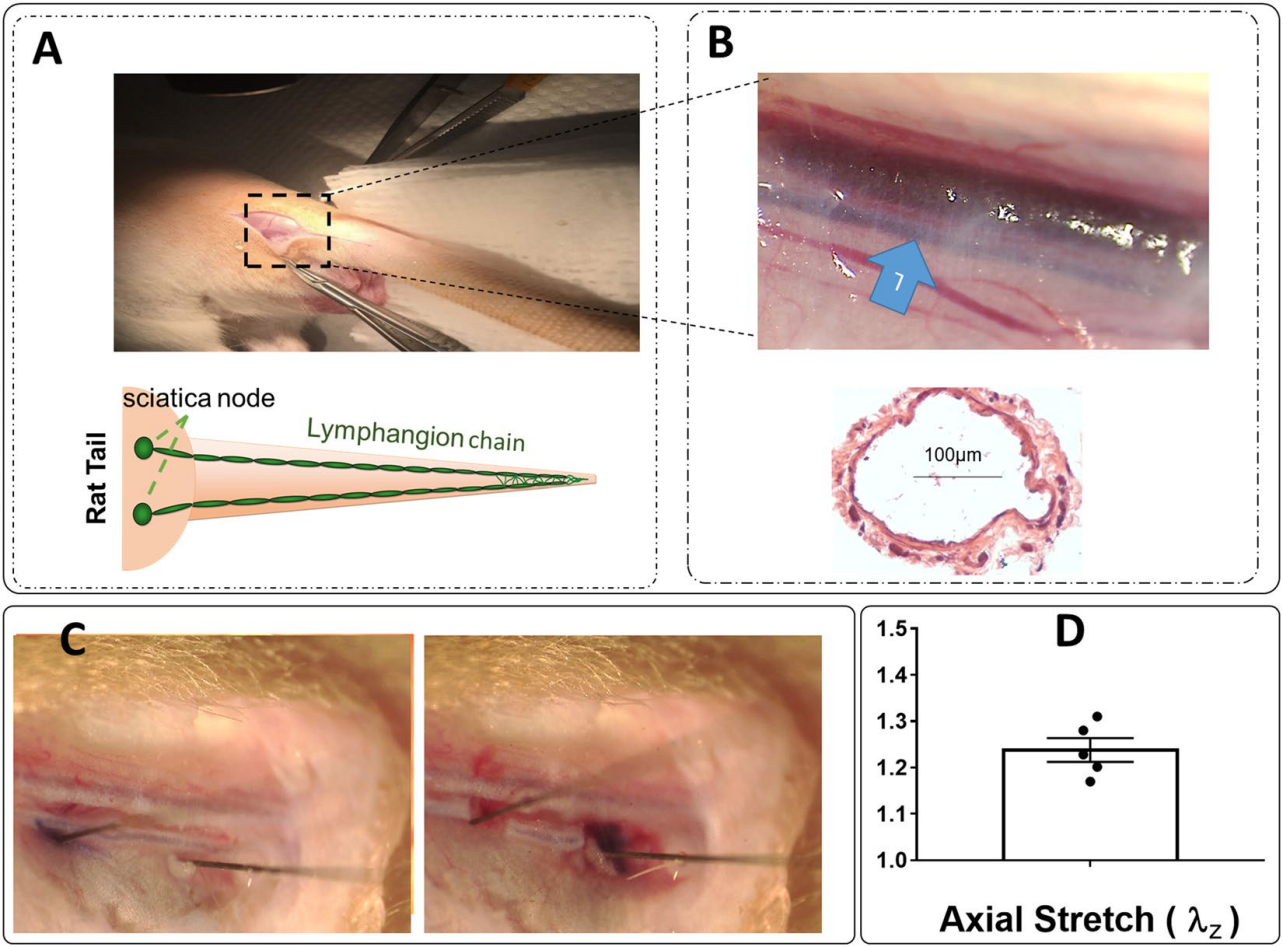
Two chains of lymphatic collectors run parallel in a rat tail (Panel A). An image of the location where an incision was made to gain access to the lymphatic vessel close to the base of tail showing that the lymphatic vessel runs parallel to the tail vein and arteries along with an image of H&E staining for a section of vessel (Panel B), an image showing lymphatic vessels from rat tails are under axial stretch *in situ* where insect pins mark the length of vessel prior to the dissection (Panel C), measurements of *in situ* axial stretch (Panel D, n = 5) where, each point represents a measurement in a different animal, are provided.

All animal experiments were approved by Georgia Institute of Technology’s Institutional Animal Care and Use Committee and were performed in accordance with the principles outlined in the National Institutes of Health, Guide for the Care and Use of Laboratory Animals. A ~1-cm incision was made on one side of the rat tail, near the base, to gain access to the lateral tail vein of male Sprague Dawley rats (300–350 g), with care taken to avoid injury to the lateral vein or artery (Fig. 1A). Trypan Blue Solution 0.4% dye was injected upstream of the incision site to enhance the visibility of lymphatic vessels (Fig. 1B). Under a stereo microscope, a lymphatic chain that runs parallel to the lateral vein was visualized.

### Quantification of *in situ* axial stretch

To approximate the *in situ* axial stretch, animals (*n* = 5, one vessel per animal) were placed under a ZEISS dissecting microscope equipped with an AxioCam MRc 5 (Zeiss, Inc.) camera. After removing the perivascular tissue, pins (0.2 mm diameter, Fine Science Tools, Inc.) were used to mark the length of the segment at the dissection locations (Fig. 1C). Using microsurgical scissors, the lymphatic vessel was dissected at the two pins (Fig. 1C). Digital images were acquired before and after excision and ImageJ software was used to determine the pre- and post-excision length; the *in situ* axial stretch was defined as ${\lambda }_{z,insitu}={\ell }_{insitu}/{L}_{u}$, where ${\ell }_{insitu}$ is the pre-excision length and ${L}_{u}$ is the post-excision, unloaded length.

### *Ex vivo* experimental setup, protocol, and data acquisition

**Figure 2:**
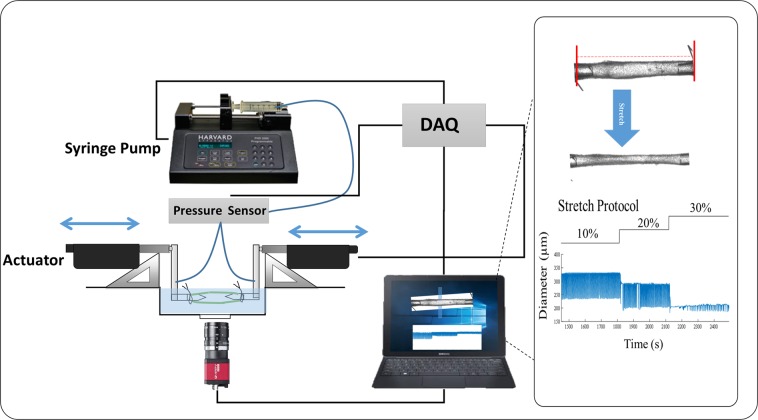
A schematic diagram of *ex-vivo* measurement of axial stretch using *ex vivo* setup. The vessel was pressurized using a syringe pump at ~0.5 mmHg to determine the minimum length at which the vessel does not bend (unloaded length). Length was measured using a LabView program and axial stretch was determined with respect to unloaded length and actuators were used to change axial stretch. A custom-written LabView program was used to track contractions as a function of time. An image of a rat tail lymphatic vessel cannulated on glass micropipettes along with respective raw traces of contractions at different stretches are shown.

For vessels used for *ex vivo* experiments, a ~2-mm segment of the collecting lymphatic vessels (*n* = 6, one vessel per animal) was excised parallel to the lateral vein and placed in Dulbecco’s Phosphate Buffered Saline (DPBS, Corning) for the removal of the surrounding fat and connective tissues. The excised and cleaned lymphatic vessel (containing approximately 1–2 valves) was mounted on opposing cannulae in a custom-made vessel chamber^33^. A custom biomechanical testing device, with some modifications^33–35^, was employed to control transmural pressure and axial stretch, while monitoring diameter. Transmural pressure was adjusted using a feedback loop with a syringe pump (PHD 4400, Harvard Apparatus, Inc.) and pressure sensors (1 psi SSC series, Honeywell). Axial stretch was controlled via computer-controlled linear actuators and XYZ stages (Newport Precision, LTA series and M-461 series). This system was placed on an inverted microscope (2.5x magnification) and the vessel diameter was recorded via a digital camera (Allied Vision Technologies, Marlin F-033B, at 18 fps); recordings were post-processed to obtain diameter tracings (Fig. 2).

In a temperature-controlled bath containing DMEM/F-12 (Dulbecco’s Modified Eagle Medium/Nutrient Mixture F-12 plus Pen-Strep 1:100, pH = 7.4 at 37 °C)^36^, the vessels were equilibrated at 2 mmHg for ~1 hour to develop stable, repeatable contractions. For the axial stretch protocol, vessels were preconditioned by delivering three pressure steps (2 mmHg, 4 mmHg, 6 mmHg) over ~5 minutes before the pressure was held constant at 2 mmHg; this pressure was chosen based on a pilot study to determine the pressure at which contraction amplitude is maximum. The unloaded length ($$L)$$ was defined as the minimum axial length required to the keep vessel straight at the (nearly) zero pressure; i.e., the pressure just above the pressure at which the vessel collapses, ~0–0.5 mmHg. The vessel length was adjusted using the precision micromanipulators to determine the length at which the vessel starts to bend. This length ($$L)$$ was measured as the distance between the two mounting sutures on glass pipettes, while viewing the vessel on the inverted microscope, via the digital camera, with a custom-written LabView code. The axial length of the vessel was increased so that the axial stretch ($${\lambda }_{z}=\ell /L$$, where $$\ell $$ is the loaded axial length, defined as the distance between the two sutures after stretching) was 10%, 20%, and 30% of the unloaded length. Vessels were held at each axial stretch for 5 minutes and contraction data collected during this time were recorded and analyzed. Based on our preliminary experiments and to prevent damage and plastic deformation that may occur during supra-physiological loading and would alter the unloaded length, diameter, and the overall response of subsequent cycles, we limited our experiments to a single pressure of 2 mmHg.

### *Ex vivo* contractility metrics and statistics

A custom-written MATLAB (MathWorks) program was used to calculate end-systolic diameter (ESD), end-diastolic diameter (EDD), and contraction frequency (FREQ) based on the raw data obtained during experiments. From these raw measurements, contractile amplitude (AMP), ejection fraction (EF), and fractional pump flow (FPF) were calculated as:

1 $${\rm{Amplitude}}:\,({\rm{AMP}})=({\rm{EDD}}-{\rm{ESD}})$$

2 $${\rm{Ejection}}\,{\rm{fraction}}:({\rm{EF}})=[{{\rm{EDD}}}^{2}-{{\rm{ESD}}}^{2}]/{{\rm{EDD}}}^{2}$$

3 $${\rm{Fractional}}\,{\rm{pump}}\,{\rm{flow}}:({\rm{FPF}})={\rm{EF}}\times {\rm{FREQ}}$$

### Confocal microscopy for collagen organization

Multiphoton confocal images of unfixed rat tail collecting lymphatic vessels, loaded with a transmural pressure ($p$ = 2 or 8 mmHg) and ${\lambda }_{z}$ = 10% on our custom mechanical testing device, were acquired using a Zeiss LSM 710 system with 40×/1.3 and Plan-Apochromat 63×/1.4 oil-immersion objectives. The second harmonic generation (SHG) of collagen fibers were obtained under 800 nm excitation wavelength. To compare differences in collagen organization, image stacks were collected in the valvular and tubular regions; the tubular region was defined as 100 µm distance from the valve. Note that a typical rat tail is 3.3 mm^37^. The image z-stacks were imported in ZEN lite (Zeiss, Inc.) software to obtain 3D reconstructions. Quantitative parameters, including fiber angle and fiber straightness, were obtained using CT_FIRE software (downloaded at https://loci.wisc.edu/software/ctfire) that automatically extracts individual fibers by applying the curvelet denoising filter followed by an automated fiber extraction method^38,39^. CT_Fire uses a two-step approach i) a curvelet filter is applied to denoise the image and enhance fibers edges ii) a fiber tracking algorithm (FIRE) detects fiber boundaries and center points and calculates length of fiber, angle and straightness. Straightness ranges from 0 to 1 and is defined as the linear length of the fiber divided by the distance along the fiber.

### Whole-mount immunofluorescence staining for LMC organization

Rat tail lymphatic vessels were isolated and fixed, unloaded, for two hours in 4% PFA at room temperature. After fixing, the vessel was washed three times with PBS and blocked with 5% goat serum in PBS and 0.5% Triton X-100 for 1–2 hours, followed by addition of the primary antibody (1:100; α-SMA Sigma) and incubation for 48 hours overnight at 4 °C (primary antibody was replaced with fresh antibody after 24 hours). After washing in PBSTx (0.3% Triton X-100 in PBS) three times, the sample was incubated with the secondary antibody at room temperature for 2 hours (1:1000, Alexa Fluor® 488, goat anti-mouse antibody). After finishing the staining, the vessel was washed in PBSTx three times and mounted in Prolong Gold for imaging. The orientation of the nuclei was obtained from DAPI staining and analyzing the respective images with CellProfiler software^40^.

### Statistical analysis

Each experimental data point represents data from a unique animal. To determine statistical significance, a one-way ANOVA, with Bonferroni’s post-hoc correction, was used to make multiple comparisons. Prism 5 (GraphPad Software) was used to perform all statistical analyses, with *p* < 0.05 considered as statistically significant, and results were reported as means (±SEM).

## Results

### Rat tail lymphatic vessels are under axial stretch *in situ* and increased axial stretch reduces pumping function

**Figure 3:**
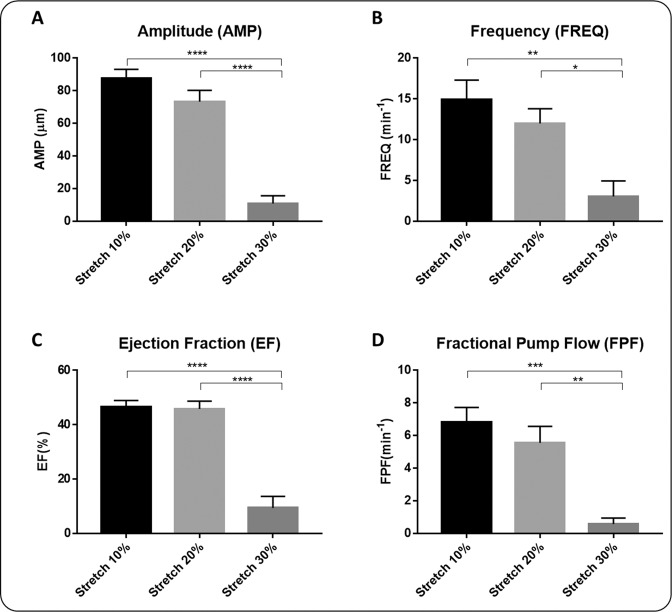
Effect of axial stretch on contractile function of lymphatic vessels. The contractile parameters were plotted as a function of the axial stretch (n = 6), including amplitude of contractions (AMP; Panel A), frequency of contractions (FREQ; Panel B), ejection fraction (EF; Panel C), and fractional pump flow (FPF; Panel D). The pressure was set to 2 mmHg for all experiments. All data are presented as mean (±SE) and statistical significance was tested via ANOVA and Bonferroni post-hoc tests (*p < 0.05, **p < 0.005).

The measured *in situ* axial stretch, ${\lambda }_{insitu}=1.24\pm 0.03$ (*n* = 5), demonstrates that rat tail lymphatic vessels are under significant axial stretch (Fig. 1). Representative diameter tracings illustrate the changes in the contractile response of rat tail collecting lymphatics under constant pressure, *ex vivo*, with sustained changes in axial stretch (Fig. 2). *Ex vivo* experiments on the isolated vessels (n = 6) showed that the amplitude of contractions decreased as the stretch increased, with significant reduction (50 µm equal to ~90% reduction) at 30% axial stretch, compared to 10% stretch (Fig. 3). Similar trends were observed for the frequency, which decreased from 15 min^−1^ to <5 min^−1^ (~80% reduction), the ejection fraction, which decreased from 46% to 9% (~80% reduction), and the fractional pump flow, which decreased from 6.8 min^−1^ to 0.58 min^−1^ (~92% reduction) from 10% to 30% stretch. Notably, these metrics of contractions changed significantly between 20% and 30% contraction, which represents the range of axial stretches measured *in situ*. Taken together, end systolic diameter, end diastolic diameter, frequency, contractile amplitude, ejection fraction, and fractional pump flow all decreased with increasing axial stretch, predominantly from 10% and 20% to 30% stretch, demonstrating that contractile function of rat tail collecting lymphatic vessels are sensitive to changes in axial stretch.

### Collagen fibers are predominantly oriented in the axial direction

**Figure 4:**
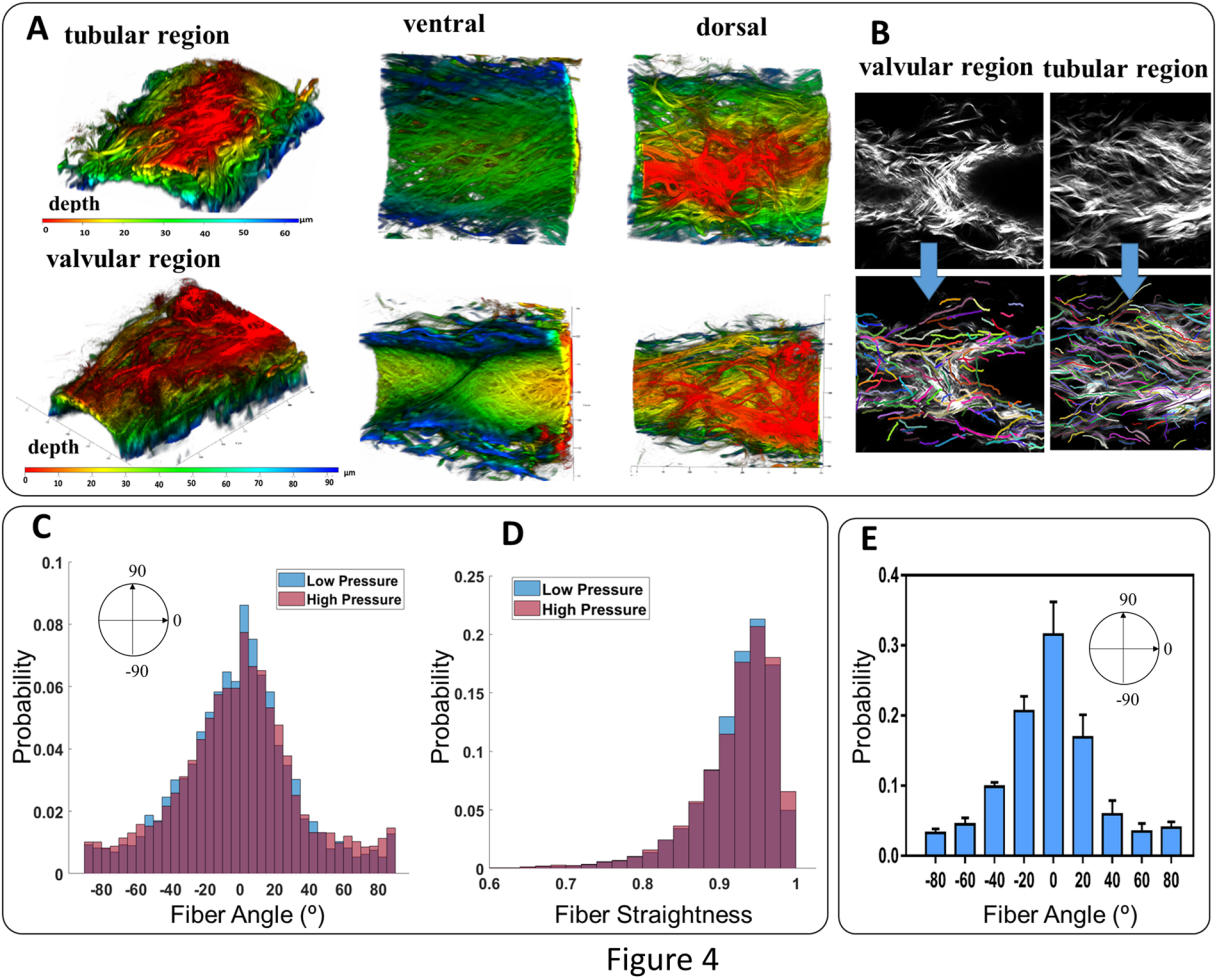
Collagen fiber organization within the wall of a pressurized, unfixed rat tail lymphatic vessel in tubular and valvular regions including reconstruction of SHG imaging of collagen fibers along with dorsal view ventral views (Panel A), the z-stack images obtained from SHG imaging of collagen fibers and respective fiber extractions using CT-FIRE algorithm (Panel B), distribution of collagen fiber angles (Panel C), distribution of collagen fiber straightness within the vessel wall for low (~2 mmHg) and high (~8 mmHg) pressure scenarios (Panel D), and the average distribution of collagen fibers (*n* = 5) plotted as the mean value along with the standard deviation (Panel E, the width of each bin is 20° and values of *x-axis* indicate the center of each bin). The vessels were excised from the base of the tail and mounted on glass pipettes in a custom-made vessel chamber compatible with the microscope. The vessels were unfixed, the pressure was ~2 mmHg, and the axial stretch was ~10%. The axial direction corresponds to 0° and the circumferential direction corresponds to ±90°.

A typical 3-D reconstruction of a vessel, based on the z-stack of images, illustrates predominantly axially oriented collagen fibers, both in the tubular region and valvular region of the rat tail collecting lymphatics (Fig. 4). The CT_FIRE code analysis of collagen fibers in the tubular region showed that the probability distribution for fiber angle (*n*=5) was maximum in the axial direction (defined as 0°) and the minimum of probability distribution occurred at ~±75°. Further, imaging of collagen fiber orientation at high pressure (p ≅ 8 mmHg), compared to those at lower pressure (p ≅ 2 mmHg) in tubular regions, revealed that an increase of pressure does not change the peak fiber angle of collagen, which was in the axial direction; however, the circumferential fiber angle increased (<5%) and fiber straightness increased suggesting a small percentage of collagen fibers are recruited at high pressures. At physiological pressure, the distribution was normal with a maximum near the axial direction (~70% of fibers, −30 < α < 30).

### LMCs are oriented in both the circumferential and axial directions

**Figure 5:**
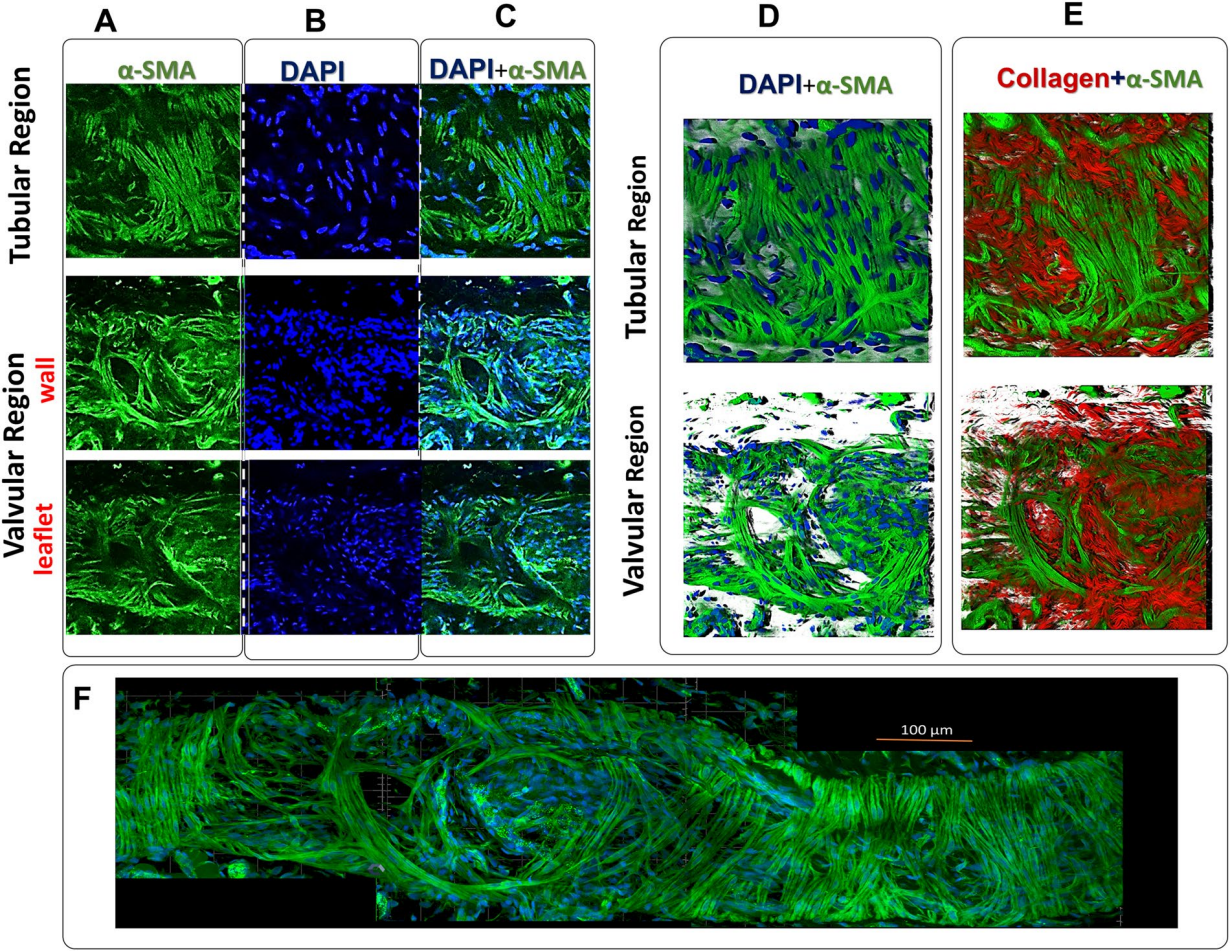
Lymphatic smooth muscle coverage and fiber organization in collecting lymphatic vessel from rat tails. A representative image (z-stack) staining for smooth muscle actin (α-SMA; Panel A), nuclear staining (DAPI; Panel B), and merged SMA and DAPI (Panel C) for valvular and tubular regions of a lymphatic vessel from rat tails. The 3D reconstruction of actin fibers of LMCs with nuclear staining (DAPI and SMA; Panel D) and with collagen fibers were obtained from SHG imaging (collagen and SMA; Panel E). The vessel was fixed and placed on a glass slide for imaging. The images were taken from the same vessel and the imaging window dimensions and depth were ~300 µm × 300 µm and ~10 µm respectively. The images from different locations were stitched together to obtain an image of the vessel (Panel F).

**Figure 6:**
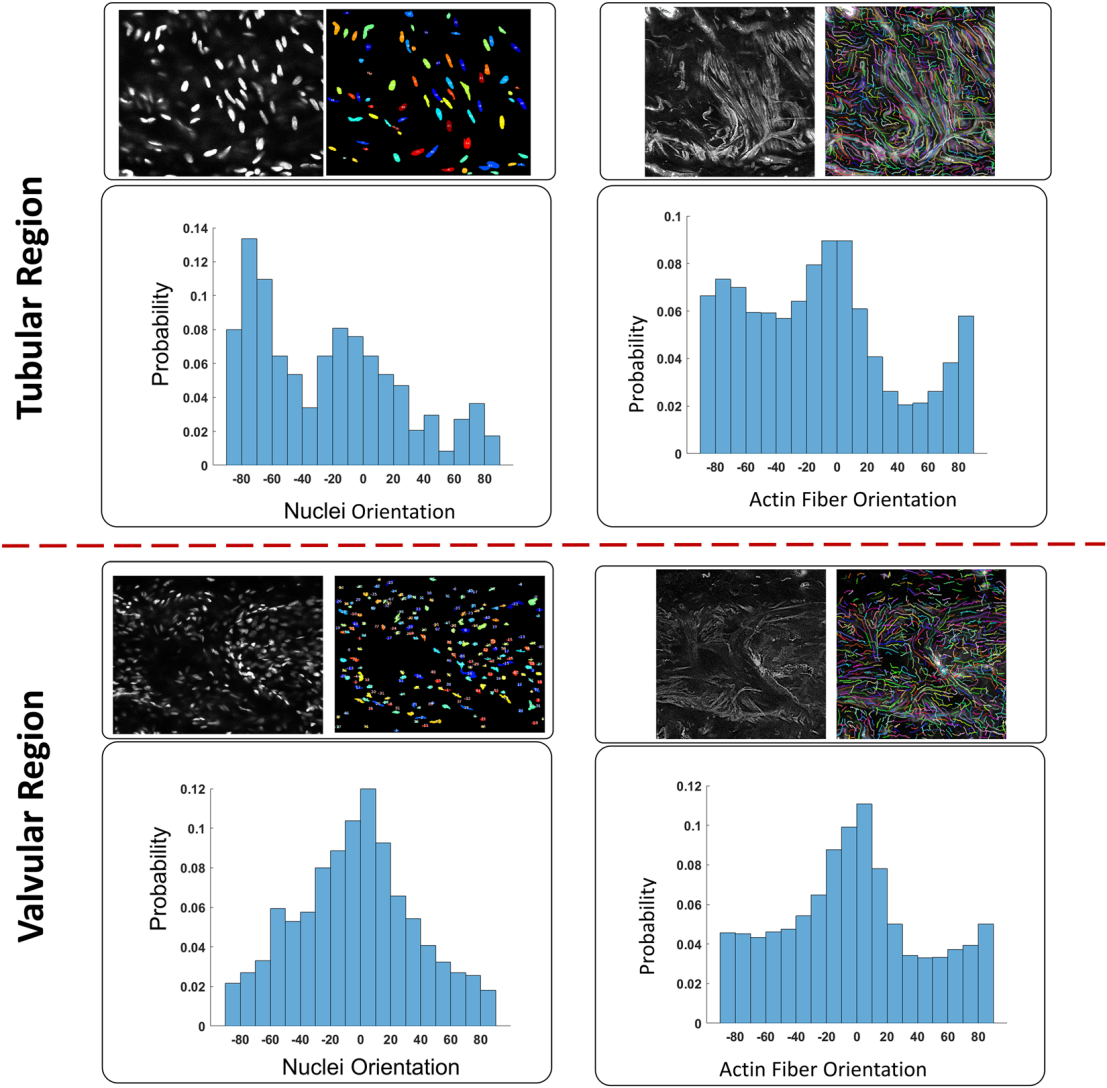
Histograms of nuclei obtained for tubular and valvular regions (*n* = 5) from DAPI staining represented in Fig. 5. The axial direction corresponds to 0° and the circumferential direction corresponds to ±90°.

Whole mount α-SMA and DAPI staining revealed a dense single layer of LMCs; occasionally, a second layer of LMC’s was observed (Figs. 5 and 6). Both DAPI and actin images illustrate that LMC bundles in the tubular region were predominantly oriented in the circumferential direction, with a second, smaller, more sporadic subset of LMC bundles oriented in the axial direction. In contrast, LMCs in the valvular region were more randomly organized, with thick bundles of LMCs primarily in the longitudinal direction. The analysis of nuclei orientation (*n* = 5) confirmed that most cells, as well as collagen fibers in the valvular region, were aligned in the axial direction, whereas the tubular region’s nuclei distributions were circumferential and axial. Taken together, the local maximum of nuclei and actin fiber orientations occurred in both axial and circumferential directions (Figs. 5 and 6), but collagen fibers were predominantly oriented in the axial direction (Fig. 4).

## Discussion

This study shows that rat tail collecting lymphatic vessels are under both circumferential and axial loading, under physiological conditions, and that changes in axial stretch have a significant effect on the contractile pumping function of lymphatics. These results suggest that axial stretch may play an important role in regulating lymph transport and changes in axial strains could be an important factor in disease progression. Further, these results reveal an important consideration that lymphatic experimentalist must consider when quantifying lymphatic contractile function; namely, consistency in axial strain is important when comparing results across experiments and across research groups.

### Axial loading likely varies over the pumping cycle

**Figure 7:**
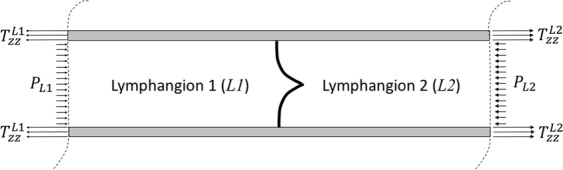
Illustrative schematic of two adjacent lymphangions, separated by a one-way valve. The trans-valvular pressure (e.g., $${P}_{L2}-{P}_{L1}$$) will lead to differences in the Cauchy stress in the wall. Differences in the axial component of the Cauchy stress requires differences (e.g., $${T}_{zz}^{L2}-{T}_{zz}^{L1})$$ necessitate differences in the axial stretches ($${\lambda }_{z}^{L1}$$ versus $${\lambda }_{z}^{L2}$$). This simple mechanical analysis suggests that axial stretch in a lymphangion will change over the pumping cycle, as valves open and close and lymphangions contract differentially in time.

Simple mechanical analysis shows that over a typical pumping cycle, the axial stress and stretch in the lymphatic wall may change significantly. Static equilibrium of two adjacent lymphangions (Fig. 7) requires that

4 $${T}_{zz}^{L2}{A}_{w}^{L2}-{T}_{zz}^{L1}{A}_{w}^{L1}={P}_{L2}{A}_{\ell }^{L2}-{P}_{L1}{A}_{\ell }^{L1}$$

where ${T}_{zz}^{L1}$ and ${T}_{zz}^{L2}$ are the axial components of the Cauchy stress, ${A}_{w}^{L1}$ and ${A}_{w}^{L2}$ are the cross-sectional area of the vessel wall, ${A}_{\ell }^{L1}$ and ${A}_{\ell }^{L2}\,$are the luminal cross-sectional area, and ${P}_{L1}$ and ${P}_{L2}$ are the luminal pressure in lymphangion 1 and lymphangion 2, respectively. For an elastic lymphatic wall, ${T}_{zz}^{L1}$ and ${T}_{zz}^{L2}$ are a one-to-one function of the in-plane circumferential and axial stretches, ${\lambda }_{\theta }^{L1}$ and ${\lambda }_{z}^{L1}$ and ${\lambda }_{\theta }^{L2}$ and ${\lambda }_{z}^{L2}$, respectively, where ${\lambda }_{\theta }^{L1}={r}^{L1}/{R}^{L1}$ and ${\lambda }_{z}^{L1}={\ell }^{L1}/{L}^{L1}$ where ${R}^{L1}$ and ${R}^{L1}$ are the loaded and unloaded radii of $L1$ and ${\ell }^{L1}$ and ${L}^{L1}$ are the loaded and unloaded lengths of $L1$, respectively. Consider a simple case where ${A}_{\ell }^{L1}={A}_{\ell }^{L2}=\pi {r}_{i}^{2}$, and ${A}_{w}^{L1}={A}_{w}^{L2}=\pi ({r}_{o}^{2}-{r}_{i}^{2})\approx 2{r}_{i}h$, where ${r}_{i}$ and ${r}_{o}$ are the luminal and outer radius and $h$ is the wall thickness. For this simple case,

5 $${T}_{zz}^{L2}-{T}_{zz}^{L1}=({P}_{L2}-{P}_{L1})\frac{{r}_{i}}{2h}$$

which says that, for a difference in transmural pressure $({P}_{L2}-{P}_{L1})\,$across the valve between two lymphangions, the axial stress (and stretch) will be greater in the lymphangion under higher pressure and lower in the lymphangion under lower pressure, due to the differential pressure $({P}_{L2}-{P}_{L1})\,$applied across the closed valve. This differential pressure increases the stretch in $L2$ and decreases the stretch in $L1$. This analysis suggests that throughout the pumping cycle there is likely significant relative changes in axial stress (${T}_{zz}^{L1}$ versus ${T}_{zz}^{L2}$) that arises due to transvalvular pressure differences caused by the combination of contraction and the opening and closing of valves. Of course, throughout pumping, as the vessel contracts and dilates, the cross-sectional areas of the lumen and wall also change differently in time between the two lymphangions, which in turn alter the axial stress and stretch of each lymphangion. Further, the lymphangions that are proximal and distal to $L1$ and $L2$ also affect ${T}_{zz}^{L1}$ and ${T}_{zz}^{L2}$. We have observed significant axial motion of isolated lymphangions, in both *in situ* and *ex vivo* settings over the pumping cycle, supporting these analyses (data not shown).

We observed that the stretch that yields the maximum contractile response is below the measured *in situ* axial stretch. It should first be noted that the *in situ* axial stretch and the *ex vivo* axial stretch may not be directly comparable measurements; e.g., the *ex vivo* measurements were made after mechanical preconditioning and isolation in a bathing solution of PSS, whereas the *in situ* measurements were made while the vessel was under *in vivo* loading, with lymph fluid in the lumen, but bathed in PSS, without any mechanical preconditioning. Methodological differences aside, given that the axial stretch likely changes throughout the pumping cycle, the observation that maximum pumping efficiency occurs at stretches below the mean *in situ* stretch may suggest that these cyclic changes in axial stretch could play a role in regulating and even orchestrating timing of contractions; e.g., as the axial stretch in a lymphangion decreases over the pumping cycle, perhaps this triggers that lymphangion to initiate contraction. Similarly, as the axial stretch increases over the pumping cycle, perhaps this triggers the LMCs to relax.

Equation (4) states that, for equivalent radii in $L1$ and of $L2$ (and thus, ${\lambda }_{\theta }^{L1}={\lambda }_{\theta }^{L2}$), the axial stretches in each lymphangion, ${\lambda }_{z}^{L1}$ and ${\lambda }_{z}^{L2}$, are governed by the constitutive relation (e.g., strain-energy function) that relates stress to strain (or stretch) for the vessel wall. For a stiffer material, the differences in axial stretch (${\lambda }_{z}^{L1}$ versus ${\lambda }_{z}^{L2}$),for a given geometry and given difference in pressure will be smaller, compared to that of a more compliant vessel. Anisotropy, too, will play an important role in controlling differences in the circumferential and axial stiffnesses. Therefore, it is important to quantify the biaxial mechanical behavior of lymphatics and the microstructural content and organization to quantify material behavior and anisotropy; see Caulk *et al*.^33^.

### Collagen fibers are orientated primarily in the axial direction, suggesting material anisotropy and increased stiffness in the axial direction

Collagen fibers play a key role in structural support and mechanics of lymphatic vessels. The mechanical properties of a load bearing tissues depend on the anisotropic architecture of collagen fibers, with the predominant collagen fiber direction correlating to increased stiffness in that direction. The current observation suggest that the predominant axial orientation of collagen fibers provide structural support for axial loads due to *in vivo* axial stretch, perhaps serving to regulate change in axial stretch throughout the pumping cycle. Collagen organization and fiber recruitment are important factors that not only determine the passive function but also influence the contractile function of lymphatic vessels. Recent multiphoton imaging of collagen fibers within the wall of the pelvic lymphatic vessel has shown that the fibers are predominately oriented in the axial direction^41^. Similar results were found for the bovine^42^ and rat lymphatics, although there are some variations^33^. Data on lymphatic vessel biaxial mechanics and compositions are limited to a few measurements from rat thoracic duct, rat mesentery, and human pelvis^33,41,43^. Observations from rat thoracic duct have shown the non-linear response and vessel stiffening occurs in response to both axial stretch and circumferential stretch^33^. It is believed that collagen, elastin, and LMCs are the main structurally-significant components that constitute the mechanical behavior of lymphatic vessels^33,41–43^.

### Axially-oriented LMCs may contribute to axial-oriented contractions and changes in axial stretch

Another way that axial stretch may change over the pumping cycle is through the direct contraction of LMC’s oriented in the axial direction. Ohtani and Ohtani, report α-SMA positive cells in rat diaphragm run longitudinally between intraluminal valves, but circumferentially in the valve regions^44^. In guinea pig mesenteric lymphatics, von der Weid showed that some LMCs are longitudinally orientated while others are orientated at approximately a 45° angle^45^. An investigation of lymphatic vessels in the diaphragm, Moriondo *et al*. suggest that active vessels showing rhythmic contractions have a dense mesh of actin fibers, but non-contracting vessels have longitudinal actin fibers^46^. F-actin staining in human thoracic ducts has suggested that non-contracting thoracic vessels have an irregular organization of lymphatic LMCs and muscle bundles are heterogeneously oriented in the circular, oblique, and longitudinal directions^47^. Micrograph observation in monkeys has revealed cervical sections of the thoracic duct are less muscular compared to the abdominal and thoracic sections, suggesting the passive function of the cervical part^48^. In the current study, immunostaining and imaging of fibers revealed that the orientation of lymphatic LMCs is heterogeneous. In the tubular region, most fibers have circular orientation, with a subset of longitudinally-oriented LMCs. In the valvular region, actin fibers were predominantly in the axial orientation.

One limitation of this study is the fact that *ex vivo* and *in situ* axial stretch measurements are not quite comparable. To determine *in vivo* stretch, vessels were cleaned *in situ* and then stretch was quantified as described in the Methods section. In contrast, to determine *ex vivo* stretch, vessels were dissected, cleaned first, and subsequently mounted in a vessel chamber. Once preconditioned in temperature-controlled conditions, the stretch was calculated with respect to unloaded length. Further, the goal of *ex vivo* experiments was to study the effect of axial stretch on the contractions in a pressure-controlled condition. while lymphatics are mechanically supported by the surrounding perivascular tissue *in vivo*, *ex vivo* testing requires lymphatic vessels to be isolated from this perivascular support. While *ex vivo* experiments have the benefit of ‘isolating’ the observed mechanical response to the tissue of interest alone, thereby removing other confounding factors that may arise due to the perivascular tissue, the disadvantage is that the applied loading, *ex vivo*, does not simulate the exact *in vivo* loading condition delivered from the perivascular tissue.

Another limitation of this study is that we did not measure changes in axial force with changes in axial stretch. Our group has previously measured the axial force of lymphatic vessels during passive mechanical testing^33^. The passive axial forces under physiological pressure and axial stretch are very small (~0.1 mN) and differences in axial force due to contractions is, perhaps, an order of magnitude lower than the passive axial force. While it is possible to resolve such differences over acute mechanical testing (over a few minutes) with our experimental device, over longer experiments of 20–30 minutes, even the highest resolution load cells experience a drift on the order of 10^th^’s or 100^th^’s of mN’s. Therefore, we could not, with confidence, report nominal values for measured changes in axial force versus changes in contractile function.

In closure, this work shows that the contractile function of rat tail collecting lymphatics are sensitive to changes in axial stretch. The microstructural organization of these lymphatics show that collagen fibers are aligned predominantly in the axial direction and a subset of LMCs are also axially-aligned, both of which may serve as a conduit for this mechanical signal (axial stretch) to be transduced to a biological response (altered contractility). These findings highlight the important role of axial loading in lymphatic pumping function, which may play a role in lymphatic disease.
